# Status of complete proteome analysis by mass spectrometry: SILAC labeled yeast as a model system

**DOI:** 10.1186/gb-2006-7-6-r50

**Published:** 2006-06-19

**Authors:** Lyris MF de Godoy, Jesper V Olsen, Gustavo A de Souza, Guoqing Li, Peter Mortensen, Matthias Mann

**Affiliations:** 1 Department of Proteomics and Signal Transduction, Max-Planck-Institute of Biochemistry, Am Klopferspitz, 82152 Martinsried, Germany; 2 Center for Experimental BioInformatics, Department of Biochemistry and Molecular Biology, University of Southern Denmark, Campusvej, 5230 Odense M, Denmark

## Abstract

**Background:**

Mass spectrometry has become a powerful tool for the analysis of large numbers of proteins in complex samples, enabling much of proteomics. Due to various analytical challenges, so far no proteome has been sequenced completely. O'Shea, Weissman and co-workers have recently determined the copy number of yeast proteins, making this proteome an excellent model system to study factors affecting coverage.

**Results:**

To probe the yeast proteome in depth and determine factors currently preventing complete analysis, we grew yeast cells, extracted proteins and separated them by one-dimensional gel electrophoresis. Peptides resulting from trypsin digestion were analyzed by liquid chromatography mass spectrometry on a linear ion trap-Fourier transform mass spectrometer with very high mass accuracy and sequencing speed. We achieved unambiguous identification of more than 2,000 proteins, including very low abundant ones. Effective dynamic range was limited to about 1,000 and effective sensitivity to about 500 femtomoles, far from the subfemtomole sensitivity possible with single proteins. We used SILAC (stable isotope labeling by amino acids in cell culture) to generate one-to-one pairs of true peptide signals and investigated if sensitivity, sequencing speed or dynamic range were limiting the analysis.

**Conclusion:**

Advanced mass spectrometry methods can unambiguously identify more than 2,000 proteins in a single proteome. Complex mixture analysis is not limited by sensitivity but by a combination of dynamic range (high abundance peptides preventing sequencing of low abundance ones) and by effective sequencing speed. Substantially increased coverage of the yeast proteome appears feasible with further development in software and instrumentation.

## Abstract 2

A mass spectrometry analysis of the yeast proteome shows that complex mixture analysis is not limited by sensitivity but by a combination of dynamic range and by effective sequencing speed.

## Background

Technological goals of proteomics include the identification and quantification of as many proteins as possible in the proteome to be investigated [1-3]. However, despite spectacular advances in mass spectrometric technology, no cellular or microorganismal proteome has been completely sequenced yet. This has not hindered successful application of proteomics, as most biologically relevant studies have focused on functionally relevant 'subproteomes'. For example, our laboratory has been interested in protein constituents of organelles such as the nucleolus and mitochondria [4-6]. These proteomes have complexities of about a 1,000 proteins and are largely within reach of current technology. Other fruitful areas of proteomics have been the analysis of protein complexes for protein interaction studies [7,8] and the large-scale analysis of protein modifications [9], which also do not require analysis of the total proteome. However, if proteomics is to directly complement or supersede mRNA based measurements such as oligonucleotide microarrays in certain applications, it needs to be able to identify and quantify complete cellular or tissue proteomes. Furthermore, if proteomics is to be used in diagnostic applications by in-depth analysis of body fluids, even higher performance would be desirable [10].

Protein mixtures can be analyzed in different ways by mass spectrometry. The most widely used approach involves enzymatic digestion of proteins to peptides, followed by chromatographic separation of the peptides and electrospray ionization directly into the source of a mass spectrometer. The mass spectrometer acquires spectra of the eluting peptides and fragments the most abundant peptide ions in turn (tandem mass spectrometry or MS/MS). The tandem mass spectra are then searched against protein databases resulting in the identification of a large number of peptides from which a protein list is compiled. Importantly, mass spectrometric signal varies widely between different peptides even if present at the same amount, not all electrosprayed peptides are fragmented and not all fragmented peptides lead to successful identifications [11]. The finite sampling speed of peptides in data-dependent experiments has partial random character and also influences reproducibility of the final protein identification [12]. In particular, if a mass spectrum contains many highly abundant peptides, then signals of low abundance will not be selected or 'picked' for sequencing by the instrument. The overall protein coverage of the experiment is a function of the sensitivity of the mass spectrometer, its sequencing speed and its dynamics range.

**Figure 1:**
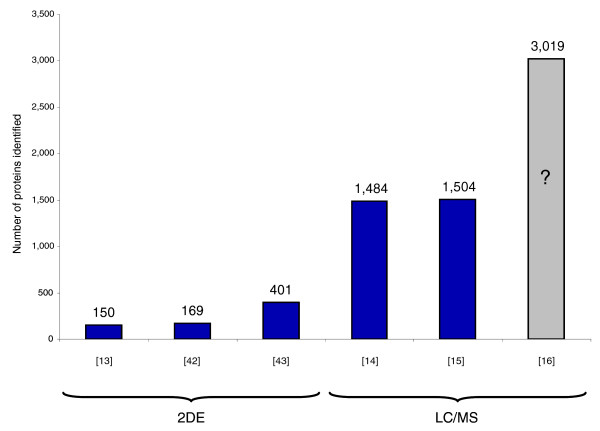
An overview of previous large-scale studies identifying yeast proteins. The studies using a combination of two-dimensional gel electrophoresis and mass spectrometry (2DE) are Shevchenko *et al*. [13], Garrels *et al*. [42] and Perrot *et al*. [43]. Experiments using only MS or 1D PAGE and MS (LC/MS) are Washburn *et al*. [14], Peng *et al*. [15] and Wei *et al*. [16]. The Wei *et al*. study is colored in grey and has a question mark because no data were provided on the identifications, making it difficult to evaluate the claim of 3,019 identified proteins, especially as low resolution mass spectrometry was employed.

Systematic elucidation of the ability of mass spectrometry-based proteomics to characterize a proteome in depth would clearly be useful, both to realistically assess current capabilities and to locate bottlenecks that should be removed. A major impediment for such studies has been the lack of a good model proteome with defined identity and abundance of the constituting proteins. The baker's yeast *Saccharomyces cerevisiae *has served as a model organism from the earliest days of proteomics, mainly to demonstrate how many proteins could be identified with a given technology (Figure 1). The first large-scale protein identification project, performed more than 10 years ago, resulted in the identification of 150 proteins [13]. Yeast was also used as the model system by Yates and co-workers [14] to illustrate their 'shotgun' and 'MudPIT' identification approaches. Those researchers and Gygi and co-workers [15] reported identification of about 1,500 proteins. A recent publication employing extensive pre-fractionation of the yeast proteome claims even higher numbers of identified proteins [16]. However, as no primary data were provided, this later claim is difficult to evaluate.

Here we make use of the data sets provided by O'Shea, Weissmann and co-workers, who have tagged each yeast gene in turn, and performed quantitative western blotting [17] as well as protein localization with GFP [18]. Their data set, for the first time, gives us both the identity and abundance of the members of a complex proteome. In logarithmically growing yeast, evidence of expression of more than 4,500 proteins was obtained, with the lowest abundance proteins at about 100 copies per cell and the most abundant proteins at about a million copies per cell. We apply state of the art mass spectrometric technologies and stringent identification criteria and show that more than 2,000 proteins can be detected in the yeast proteome by a combination of one-dimensional gel electrophoresis (1D PAGE) and on-line electrospray tandem mass spectrometry ('GeLCMS'). While proteins with very low abundance are detected, we find that the effective sensitivity in complex mixtures is orders of magnitude lower than it is for single, isolated proteins. Likewise, while the dynamic range is very high for some proteins, the average for the whole experiment is about 1,000. We employ stable isotope labeling by amino acids in cell culture (SILAC) [19] labeled yeast to investigate these limitations in effective sensitivity and dynamic range and suggest ways to improve complex mixture analysis.

## Results and discussion

### Sampling the yeast proteome by GeLCMS

**Figure 2:**
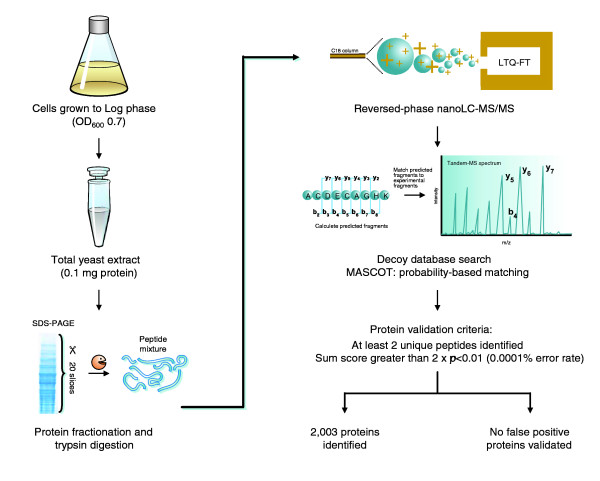
Work flow of the yeast proteomics experiment.

**Figure 3:**
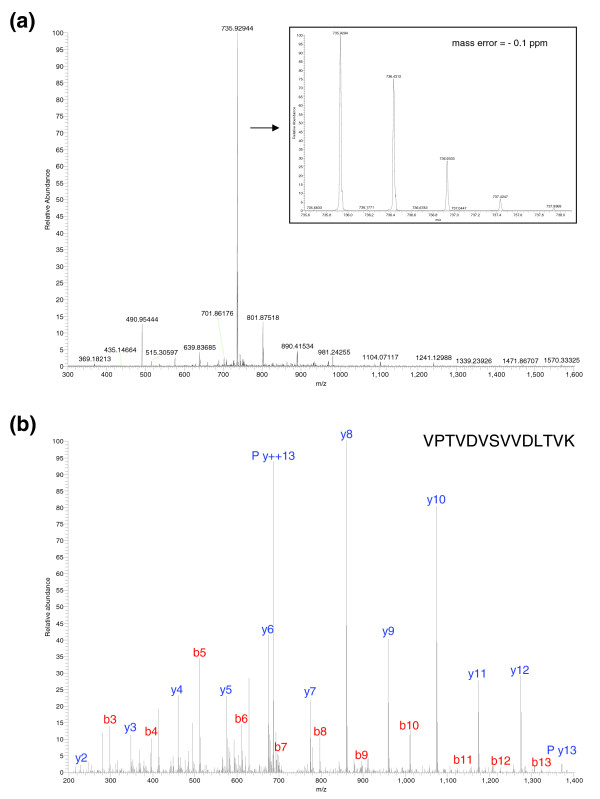
Example of MS and MS/MS on the LTQ-FT. **(a) **A mass spectrum of yeast peptides eluting from the column at a particular time point in the LC gradient and electrosprayed into the LTQ-FT mass spectrometer. The inset is a zoom of the doubly charged peptide ion at m/z 735.929, showing its natural isotope distribution and demonstrating very high resolution. **(b) **Tandem mass spectrum of the dominant peptide in (a). Peptides fragment on average once at different amide bonds, giving rise to carboxy-terminal containing y-ions or amino-terminal containing b-ions. The prominent y_13_^++ ^ion is caused by fragmentation at the first amide bond, which is favored here because it is amino-terminal to proline. (See [44] for an introduction to peptide sequencing and identification by MS.) The mass of the peptide identified is within less than 1 ppm of the calculated value.

Figure 2 is an overview of the procedure used to probe the yeast proteome. Wild-type yeast cells were grown to log-phase, lysed by boiling in SDS and 100 μg of whole cell lysate was separated by 1D PAGE. The gel was cut into 20 slices, proteins were in-gel digested with trypsin and the resulting peptides extracted from each gel slice were analyzed by automated reversed-phase nanoscale liquid chromatography (LC) coupled to tandem mass spectrometry (MS/MS). Together, the 20 LC-MS/MS runs, including intervening washing steps, lasted 48 hours. The peptides were electrosprayed into the source of a linear ion trap-Fourier transform mass spectrometer (LTQ-FT) [20]. This hybrid instrument consists of a linear ion trap (LTQ) capable of very fast and sensitive peptide sequencing combined with an ion cyclotron resonance trap (ICR). In the ICR trap, ions circle in a 7 Tesla magnetic field and their image current is detected and converted to a mass spectrum by Fourier transformation (FT-ICR). While this high resolution and high mass accuracy spectrum is acquired, the LTQ part of the mass spectrometer simultaneously isolates, fragments and obtains the MS/MS spectrum of the five most abundant peptides. These are then automatically excluded from further sequencing for 30 seconds. Figure 3a shows a mass spectrum of yeast peptides eluting at a particular time point in the LC gradient. As can be seen in the figure, mass resolution was very high (better than 50,000) and mass accuracy was better than one part per million (ppm). Figure 3b illustrates a tandem mass spectrum of the most abundant peptide in the full scan spectrum acquired by fragmentation in the linear ion trap. Because detection of tandem mass spectra happens in the linear ion trap it is highly sensitive, such that overall MS sensitivity is limited by recognition of the peptide in the full scan.

To maximize the number of ions we did not use the selected ion monitoring (SIM) scans in the FT-ICR that we had previously found to result in very high mass accuracy [21]. Instead, we operated the LTQ-FT in full sequencing mode, where full scan spectra are recorded in the ICR without acquiring SIM scans and with a high ion load (target of 5 × 10^6^) to maximize dynamic range. The high ion loads cause space-charging effects, which result in an almost constant frequency shift for all ions recorded and thereby affect mass accuracy. To correct for this shift we devised a recalibration algorithm that corrects for space charge-induced frequency errors on the basis of peptides identified in a first pass search (see Materials and methods). Using this recalibration algorithm, peptide mass accuracy improved several fold, to an average absolute mass accuracy of 2.6 ppm for our entire data set (Additional data file 1).

A total of more than 200,000 MS/MS spectra were acquired and searched against the yeast proteome using a probability based program (Mascot [22]). We first required a probability score of 15 for peptide identification, which resulted in the identification of more than 60,000 peptides, among which 20,893 represent unique sequences (Table 1; Additional data file 1; peptides will be submitted to the open archive termed Peptide Atlas [23] as well as to the PRIDE proteomics database [24]). For each unique sequence, therefore, on average three peptides were fragmented and identified. This was caused by repeated picking of the same peptide in the same or different runs, sequencing of different charge states, sequencing peptides with modifications such as oxidized methionine and sequencing peptides with missed tryptic cleavage sites.

**Figure 4:**
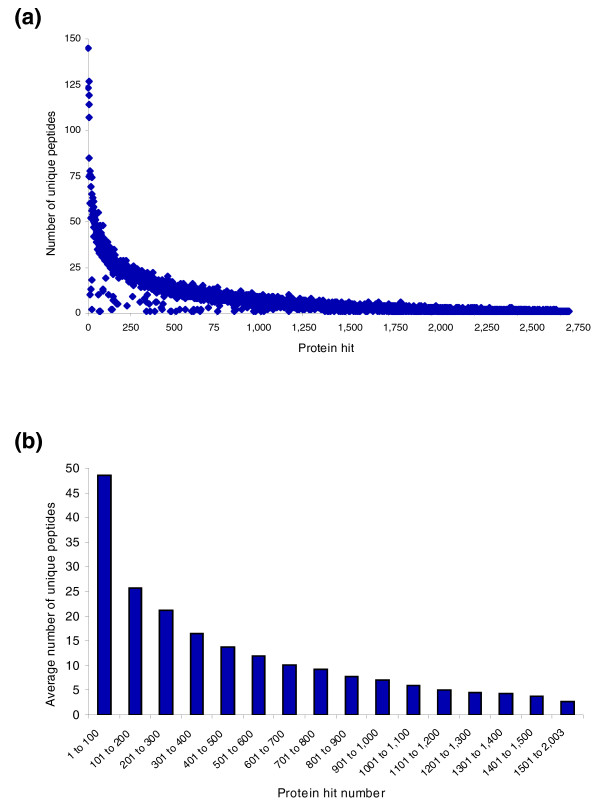
Number of peptides identifying yeast proteins. **(a) **Unique peptides with score of at least 15 and mass accuracy at least 10 ppm. Proteins are ordered by decreasing Mascot score. **(b) **Average number of unique peptides identifying proteins in bins of 100. Only peptides from verified protein hits with at least two peptides are plotted.

We next analyzed the distribution of peptides onto proteins. In Figure 4a, proteins are listed according to decreasing Mascot protein score and the number of unique peptides with a probability score of at least 15 is plotted. (Note that these are protein hits before validation.) Six yeast proteins were identified with more than one hundred peptides each and a steady decline in the number of peptides identifying each protein can be observed.

To establish criteria for unambiguous protein identification, we first noted that the probability score for 99% significance (*p *< 0.01) was 29 for these experiments. Only peptides with scores higher than 15 were considered in the analysis and a minimum of two unique peptides and a combined score of 59 were required for protein validation. The value of 59 was chosen because it corresponds to the summed score of two peptides with *p *< 0.01. Formally, if the two peptide identifications are statistically independent, a combined score of 59 would represent less than one false positive in 10,000. However, as we cover a substantial part of the yeast proteome, the probability of protein identification is a more complicated function of peptide identification [25-27]. We therefore tested our false positive rates directly in a 'decoy database' [15,28] consisting of both forward and reversed ('nonsense') yeast sequences. Peptides that are found in the reversed but not in the forward database are assumed to be false positive peptide matches. When requiring the stringent criteria outlined above, we found no false positive protein hits in the reversed database. We therefore conclude that our search criteria exclude essentially all false positives.

A total of 2,003 proteins were identified, with an average of 10 unique, verified peptides per protein. Thus, it is possible to unambiguously identify more than 2,000 yeast proteins in a single experiment involving a measurement time of about 48 hours. Almost all of the top 1,500 proteins are represented by at least three peptides (Figure 4b). We compared these results with previous proteomic studies that had been performed with the technology available a few years ago (Table 1). Using 1.4 mg of yeast lysate and three MudPIT experiments, Yates and co-workers [14] identified 848 proteins with more than one peptide and Gygi and co-workers [15] identified 991 proteins with more than one peptide and using 1 mg of cell lysate. Note that these peptides were not required to be fully tryptic and that the ion trap instruments used in those studies measured mass about a hundred times less precisely than what we reach with the LTQ-FT. Thus, this comparison is only meant to illustrate the advance in technology during the last few years, not to compare specific protein or peptide purification strategies in large-scale proteomics.

### Protein abundance versus chance of identification

**Figure 5:**
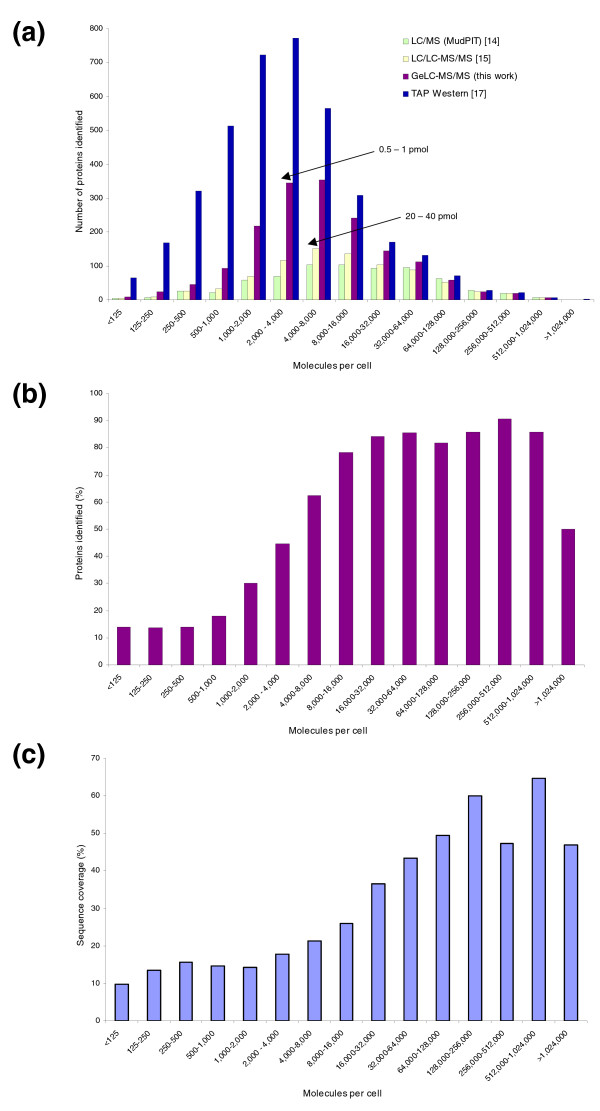
Protein abundance in the yeast proteome and identification by mass spectrometry. **(a) **Blue bars indicate the number of yeast proteins in copy number classes (recalculated from the data in Ghaemmaghami *et al*. [17]). Red bars represent the proteins identified in each copy number class in this study, green bars represent the data from Washburn *et al*. [14] and yellow bars data from Peng *et al*. [15]. The arrow labeled 0.5-1 pmol points to the bin with a 50% chance of identification (this data) whereas the arrow labeled 20-40 pmol indicates the amount and copy number needed for a 50% chance of identification by the Washburn *et al*. and Peng *et al*. studies. **(b) **Data of this study normalized to the number of proteins detected by western blotting in each copy number class. **(c) **Percentage of the total protein sequence covered by identified peptides as an average for the abundance bin. Sequence coverage for each protein is calculated in Additional data file 1.

Two recent studies of global expression [17] and localization [18] in *S. cerevisiae *were able to detect together more than 4,500 yeast proteins, indicating that at least 80% of the yeast genome is expressed in logarithmically growing cells. Using quantitative western blotting against the tandem affinity purification (TAP) tag, the authors also estimated the number of molecules per cell for 3,800 of the proteins detected. As shown in Figure 5a (blue bars), they found that yeast protein expression follows a bell-shaped curve, with an average expression of about 3,000 proteins, very few proteins at less than 125 copies and very few proteins at more than 10^6 ^copies. The dynamic range of the yeast proteome therefore appears to be about 10^4^. Also plotted in Figure 5a are the data from the two previous large-scale proteome studies (yellow and green bars) and the data from this study (red bars). As expected, due to the use of more modern mass spectrometric equipment, we were able to identify many more proteins than previous large-scale studies. Virtually all of the proteins discovered by mass spectrometry were also discovered in the TAP-tagging study independently, supporting the high stringency of protein identification in this study. More than half of the proteome for which western blotting results were available were also stringently covered by our GeLCMS approach using the LTQ-FT mass spectrometer. Interestingly, the proteins identified by MS also follow a bell-shaped curve, albeit offset by one order of magnitude to higher copy numbers.

We failed to identify some very abundant proteins. Inspection of the sequence of one of the most abundant yeast proteins (YKL096W-A), which was nevertheless not identified, revealed that it contained a single tryptic cleavage site, producing a peptide that is not readily detected by mass spectrometry. This illustrates a fundamental issue in proteomics, namely that enzymatic digestion with a single protease is likely to miss some proteins regardless of other aspects of the experiment. Conversely, some very low abundance proteins with copy number of a few hundred were also detected. In Figure 5b the mass spectrometry identification data are plotted as a percentage of total proteins in the copy number bin as detected by western blotting. In the very low abundance classes, only 10% of the proteins were identified. At a copy number of 2,000 to 4,000, the chance for identification was 50% and we used this copy number to calculate the 'effective sensitivity' and 'effective dynamic range' of this experiment, rather than the more common definition in proteomics, which is based on the lowest abundance protein that has been detected. At higher protein abundance, the chance for identification using trypsin alone climbs to more than 90%. (Note that the highest abundance class contains only two proteins, one of which is the non-detected protein discussed above.) It is clear from Figure 5 that another one to two orders of magnitude in effective sensitivity and dynamic range are needed to cover the yeast proteome completely.

It is instructive to compare these results with those for mRNA analysis, the current standard for global gene expression measurement. It is generally assumed that the complete transcriptome is covered in these experiments, provided that every transcript is represented on the chip. However, mRNA analysis also has a dynamic range challenge and, according to some reports, a large part of rare messages are not accurately detected [29]. In such situations, the coverage of the proteome and transcriptome may already be similar.

We next asked how much of the sequence of the identified yeast proteins was actually discovered in the experiment. While two peptides were sufficient for identification, Figure 4 shows that many proteins were 'covered' by a large number of peptides. We calculated the average sequence coverage per abundance bin (Figure 5c). The lowest coverage is at about 10%, going up to more than 50% at 50,000 copies per cell. To have a 50:50 chance to detect a stochiometric protein modification, about a factor 10 more material is needed compared to the effective sensitivity of the experiment. Overall, our sequence coverage using a single enzyme was 25% (Additional data file 1). Use of a second enzyme would likely increase this sequence coverage substantially.

We calculated the total amount of protein corresponding to our effective sensitivity as follows. A total of 100 μg of yeast cell lysate was used, equivalent to 1.38 × 10^8 ^yeast cells. A copy number of 3,000 then corresponds to 4 × 10^11 ^molecules or 0.7 picomoles. This position is indicated by an arrow in Figure 5a. Proteins of the lowest abundance class of 100 copies per cell are still present at about 20 femtomoles, detectable if they were single, gel-separated proteins [30]. While representing a several-fold improvement compared to previous proteomic data, protein identification in our GeLCMS experiment was thus still relatively non-sensitive when compared to the subfemtomole amounts required for detection of single proteins by mass spectrometry. This indicates that other factors, such as up front fractionation, dynamic range and sequencing speed dramatically influence the effective sensitivity in complex mixtures analysis.

### Fractionation to increase proteome coverage

The simplest analysis procedure is to digest entire proteomes and analyze them directly in a single LCMS run. They can also be fractionated at the protein level or at the peptide level before analysis. In principle, proteome coverage should be improved by any increase in the number of analyzed fractions. In this report we have chosen GeLCMS, a single protein fractionation step separating proteins by molecular weight preceding the LCMS analyses. Alternatively, in the LC-LC or MudPIT approach, two steps of separation are performed at the peptide level. Principle advantages of additional stages of fractionation are that demands on sensitivity are decreased if proportionately more material is employed. For example, about 10 times more material can be loaded in both GeLCMS and LC-LC compared to a single LCMS analysis. Likewise, demands on dynamic range and sequencing speed (see below) may be lower after fractionation. Principle disadvantages of extensive fractionation are increased measurement time (about a factor 10 per fractionation step) and increased sample consumption. Furthermore, in our hands, 1D PAGE and reversed phase peptide separation are by far the most robust and high resolution separation techniques for proteins and peptides, respectively, and it is difficult to efficiently separate proteins or peptides by additional methods. Thus the same peptides typically appear in many different fractions when extensive fractionation is used.

We compared our data to a single run with 10 μg of yeast cell lysate (data not shown) and found that GeLCMS resulted in four times more proteins identified. However, this increase was gained at the expense of loading 10 times more material and an analysis time 20 times longer than the single run. This example supports the general experience that extensive fractionation faces diminishing returns and is not an elegant method to obtain full proteome coverage (also see the dynamic range discussion below).

### Factors potentially affecting proteome coverage

**Figure 6:**
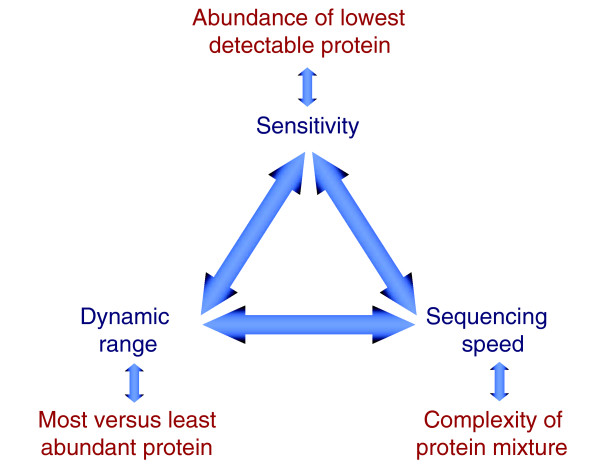
Parameters affecting the degree of proteome coverage. The dark blue terms pertain to the characteristics of the mass spectrometer and associated on-line chromatography. In red are the corresponding characteristics of the proteome. The blue arrows indicate that the three parameters are interdependent. For example, limited dynamic range and sequencing speed act together to reduce the effective sensitivity in complex mixtures to below that of single proteins.

Figure 6 depicts three instrumental factors - sensitivity, sequencing speed and dynamic range - and the corresponding proteome characteristics that together delineate the coverage of a given protein mixture in LC MS/MS analysis. Sensitivity is clearly a limiting factor if only a small amount of protein starting material is available, such as when only a few cells can be harvested in biopsies. Furthermore, if all other limiting factors are removed, then sensitivity may become the remaining barrier to complete proteome coverage. For example, if less than a femtomole of a protein of interest is present in the sample and the detection limit for this protein alone is above a femtomole, it will not be observed regardless of fractionation procedures or data acquisition strategies. Another obvious factor potentially limiting proteome coverage is the sequencing speed of the mass spectrometer [31]. Recall that the mass spectrometer is presented with many peptides at any given time as they co-elute from the chromatographic column. If the sequencing of each peptide takes longer than the average time between the appearance of new peptides, some peptides will not be sequenced even though their signal has been detected. Finally, proteome coverage can be limited by the 'dynamic range' of the instrument - the difference between the most abundant and least abundant signal in the analysis. This limitation is due to the inability of almost any measurement instrument - including mass spectrometers - to detect a very low abundance signal if a very high abundance signal is also present.

The arrows in Figure 6 indicate that these three factors interact to limit the achievable proteome coverage. For example, if there is inadequate dynamic range, low abundance components will not be recognized and, therefore, cannot be selected for sequencing, limiting effective sensitivity. Below we investigate the three parameters in turn.

#### Proteome coverage is not necessarily limited by sensitivity

Sensitivity is a key parameter in protein analysis, as there is no amplification procedure for proteins, and it would be natural to assume that proteome coverage is limited by the sensitivity of the mass analyzer. However, Figure 5 clearly shows that this is not the case in our experiments. While we identified very low abundance proteins, our effective sensitivity was about 3,000 copies per cell or 0.7 picomoles (see above). This is about a factor 1,000 lower than the sensitivity that we achieve with standard proteins with the same instrumentation [21,32]. As already noted, the least abundant yeast proteins according to Ghaemmaghami *et al*. [17] are present in about 100 copies per cell, corresponding to more than 20 femtomoles of protein, which should be detectable by our instrument. Some proteins with copy numbers of a few hundred were indeed identified in our data set. Thus, mass spectrometric sensitivity *per se *was clearly not limiting in this experiment.

#### Proteome coverage is limited by sequencing speed

##### SILAC to assess the degree of sampling in complex mixtures

To determine if proteome coverage was instead limited by sequencing speed, we first needed to distinguish true peptide peaks from chemical and electronic background. This is generally not an easy task and the mass spectrometry data system will pick peptide peaks as well as some background peaks and attempt to fragment them in the mass spectrometer (for example, see [11]). To visualize true peptide signals and to determine the degree of peptide sampling for sequencing, we used SILAC [19]. SILAC is a metabolic labeling strategy in which an essential amino acid is replaced in the media by a stable (non-radioactive) isotope analog. The proteome is labeled completely and peptides containing the labeled amino acid can be distinguished from their unlabeled counterparts in the mass spectrometer by their increased molecular weight. Although yeast can normally synthesize all amino acids, SILAC labeling is possible by using deletion strains where the synthesis pathway of the specific amino acid used for labeling is disrupted [33].

**Figure 7:**
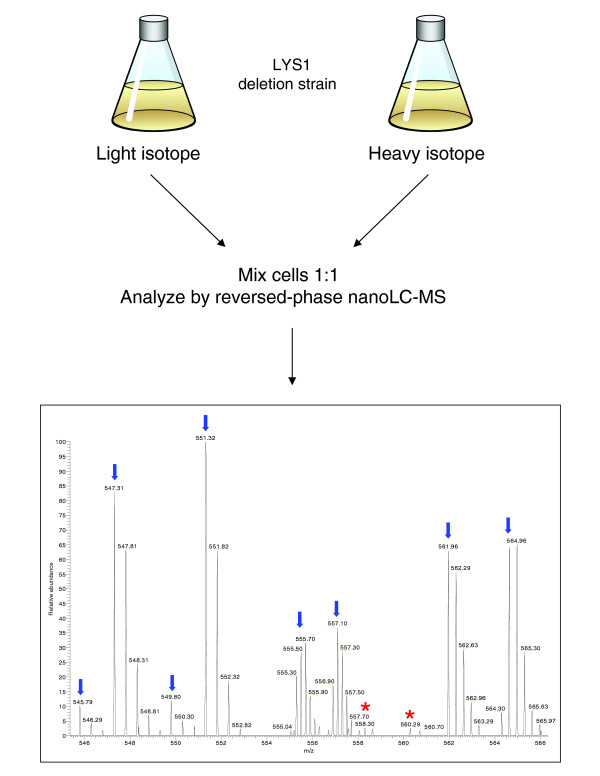
SILAC labeling of yeast to recognize true peptide signals. A yeast strain that is deficient for lysine biosynthesis is grown in the presence of normal lysine or lysine with substituted ^13^C and ^15^N, leading to a mass difference of 8 Da. Yeast cells are mixed in equal proportions, lysed, digested by endopeptidase LysC and analyzed by mass spectrometry. In the example mass spectrum, each true peptide signal is represented by a pair, spaced by 8 Da (blue arrows; mass difference appear different because peptide can have different charge states). Peaks marked by red stars are unlikely to be yeast peptides because they have no SILAC partner.

Cells were grown in defined medium containing either normal or ^13^C_6 _^15^N_2_-labeled lysine, mixed 1:1, lysed and the cell extract separated by gel electrophoresis. One of the bands was excised, in-gel digested and measured by LC MS/MS on the LTQ-FT. A flow chart of the experiment is presented in Figure 7. All peptides - except the carboxy-terminal peptide of each protein - should be present as 1:1 pairs in the mass spectra. Ideally, each SILAC pair detectable in the each mass spectrum should then be selected for sequencing and both its non-labeled ('light') and labeled ('heavy') forms should be identified. In practice, if sequencing speed is not sufficiently high, the more abundant peptide pairs will be identified in both forms, less abundant peptide pairs will be picked for sequencing in only one of the two forms and the least abundant peptide pairs may not be sequenced at all.

##### Coverage of SILAC pairs by sequencing

**Figure 8:**
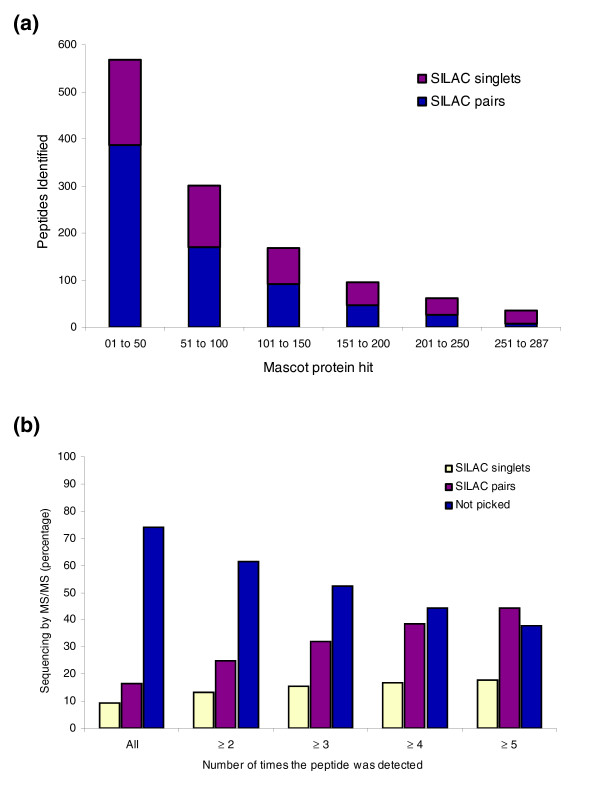
Degree of sampling of SILAC peptide pairs. Yeast was SILAC labeled as explained in Figure 7 and one gel band was analyzed. In principle, SILAC peptide pairs should both be recognized and sequenced as they are equally abundant. **(a) **Proteins identified were binned according to decreasing Mascot score. Blue bars indicate the peptide in which both members of SILAC pairs were sequenced and red bars indicate the proportion of peptides in which only one member of the SILAC pair was sequenced. **(b) **Complete analysis of the LCMS experiment for all SILAC pairs extracted by their mass differences. Peptide pairs are ordered by the number of consecutive mass spectrometry scans that they appear in, thus greater or equal than three means that the pair was detected in three or more scans.

In total, more than 1,200 unique peptides were identified in the SILAC experiment of one gel band, mapping to 287 proteins. Among these peptides, 729 were present in both heavy and light forms, while for 500 unique peptides, only one of the SILAC forms could be detected (Figure 8a). As both SILAC forms were of equal abundance, they were both recognized by the data system as candidates for sequencing. The fact that in 40% of the cases, only one of them was actually fragmented and identified shows that sequencing speed was indeed limiting. Furthermore, Figure 8a shows that SILAC pairs from abundant proteins tend to be sequenced in both forms, whereas low abundance proteins (indicated here by lower peptide number) are almost exclusively identified by sequencing of only one partner of the SILAC pairs.

To clarify this finding in more detail, we investigated the whole LC run for the occurrence of SILAC pairs, regardless of whether they were picked for sequencing or not. Using the high mass accuracy and resolution, we extracted SILAC pairs by the exact mass difference of 8.014 Da. To count as SILAC pairs, masses had to be within 10 ppm of each other (after adding the SILAC label) and both peaks needed to be accompanied by ^13^C isotopes. These criteria effectively removed noise from consideration. The list was then reduced to unique masses and SILAC pairs were classified according to the number of times they appeared in consecutive full scans. Finally, we determined for each pair whether none, one or both members of the pair were selected for sequencing. As shown in Figure 8b, for abundant peptides - those detectable in 5 or more consecutive MS scans (roughly corresponding to 20 seconds elution time) - 18% of SILAC pairs were sequenced only in one of the two states, 44% were sequenced in both forms and the remaining 38% were not sequenced at all. The low abundance peptides (those registered only for 2 consecutive scans) were not picked for sequencing in an astonishing 60% of the cases. These data show that the sequencing speed was not sufficient to fragment all recognized peptide pairs and that low abundance peaks are less likely to be sequenced than high abundance peaks. The figure suggests that, at the dynamic range achieved in this experiment, at least a factor three increase in sequencing attempts would be desirable. Any increase in dynamic range, of course, would need to be accompanied by a further increase in sequencing speed.

We note in passing that the 'effective sequencing speed' could be much higher than it is now. As observed above, in our experiment each unique sequence was sequenced and identified on average three times. Thus, if acquisition software was more intelligent in selecting peaks for sequencing, the effective sequencing speed could be at least a factor three higher, probably leading to many more identifications. Since mass accuracy is in the low ppm range, recognition of the same peptide or the same peptide in a different charge state and exclusion from further sequencing should be straightforward. Furthermore, further predicted peptides from a protein already identified with two peptides could be excluded from further sequencing, which would dramatically improve effective sequencing speed.

In principle, it would be possible that many peptides are fragmented but not identified by the search engine. However, 30% of all sequencing attempts in this experiment already led to productive identifications even at our high stringency criteria. Furthermore, reports of manual in depth analysis of high accuracy data also suggest that there is not a large fraction of proteins remaining to be identified with the aid of better peptide search engines (for example, see [34,35]).

#### Proteome coverage is limited by dynamic range

Because the yeast proteome has a dynamic range of about 10^4^, the dynamic range of the mass spectrometer ideally should be greater than this value. By inspection of mass spectra in this experiment, we found that SILAC pairs could only be identified in a range of about 100 (most abundant to least abundant pair in the same spectrum). In no case were we able to identify pairs with an abundance difference of more than a few hundred. In hindsight, this was to be expected since the FT-ICR was filled with five million charges and several hundred charges are necessary for detecting a signal. If only two species were present, then a dynamic range of 10^4 ^could be achieved. However, in our experiments, the total signal is always distributed between many peptides with different abundances, thus the effective dynamic range in a proteomics experiment is much less than the maximal dynamic range for a two component mixture.

Accumulation times for the FT-ICR full scans were set to a maximum of two seconds but typical injection times were below a hundred milliseconds. This was caused by abundant peptides that essentially determined the time it took to fill the ICR trap to the desired number of ions. Abundant proteins were present in each gel slice and abundant peptides eluted at nearly every time point; therefore, effective dynamic range was determined by the most abundant proteins. Dynamic range of the LC separation would only increase overall dynamic range if peptides were completely separated from each other rather than many peptides co-eluting at any given time. This finding also explains why additional stages of fractionation do not necessarily increase dynamic range substantially. Interestingly, purely *in silico *models have recently predicted that dynamic range is the crucial parameter in complex mixture analysis [36].

### Possible improvements in proteome coverage and perspectives for covering the entire yeast proteome

With current GeLCMS technology employing high accuracy mass measurements and fast sequencing cycles, we unambiguously cover more than 2,000 yeast proteins. There is evidence for about 1,000 additional proteins that are not listed here because their identification was ambiguous (Figure 4a). These proteins should be 'recoverable' with incremental improvements in current technology. For example, we noted that effective sequencing speed was limited by repeated sequencing of the same peptide, something that should be avoidable with better acquisition software. With these and other straightforward improvements, a protein mixture similar to the yeast proteome should be analyzable to a depth of about 3,000 proteins. Those proteins should also essentially all be quantifiable by the SILAC method, since we would obtain several quantifiable yeast peptides for the vast majority of these proteins.

Figure 5 indicates that effective sensitivity needed for the entire yeast proteome is between 10 and 100 times higher than what we achieve here and that we would need to detect about twice as many proteins. As mentioned above, mass spectrometric sensitivity is already sufficient even for the least abundant proteins.

Sequencing speed could further be improved by building a database of typically observed yeast peptides first. Subsequent identification would then be done against this peptide database and, given the very high mass accuracy that can now be obtained even on compact instruments [37], would require relatively low quality MS/MS spectra. Therefore, very fast MS/MS scans (called 'Turbo scans') could be employed, which could speed up sequencing several fold.

With these improvements, neither sensitivity nor sequencing speed would likely be limiting for the analysis of the complete yeast proteome. Dynamic range, however, can only be addressed by substantial pre-fractionation, which is an unattractive option, or by increasing the dynamic range of the mass spectrometer. Fortunately, the latter can be addressed in several ways. For example, the most abundant ions in a mass spectrum could be determined first, as is the case now. In a second accumulation of ions, these species could selectively be ejected from the ion trap allowing longer accumulation times for the remaining low abundance ions [38]. Alternatively, the total mass range can be acquired in several individual mass ranges, again allowing much longer acquisition times for the mass ranges without dominant peptides (Olsen and Mann, unpublished). By one or a combination of these techniques, it seems likely that an increase of dynamic range by at least an order of magnitude should be achievable.

## Conclusion

Here we have shown that high mass accuracy and sequencing speeds employed in state of the art proteomics can confidently identify more than 2,000 proteins in the yeast proteome without excessive fractionation and from only 100 μg of yeast cell lysate. Despite these impressive numbers, effective sensitivity in complex mixture analysis is several orders of magnitude lower than that achieved in single protein analysis. Using SILAC labeled yeast, which produces characteristic 1:1 pairs of true peptide signals, we determined that a combination of effective sequencing speed and effective dynamic range limit coverage of the yeast proteome. Our results show that current proteomics technology is indeed capable of in-depth characterizing samples containing about 1,000 to 2,000 proteins, ratifying the results obtained in previous studies of 'subproteomes' such as those of the nucleolus and mitochondria [4,5]. It also indicates in the case of more complex proteomes, such as yeast total cell lysate, only about half of the proteins expressed are being detected and the full coverage will require one to two orders of magnitude higher effective sensitivity. This can be achieved by increasing the sequencing speed by more intelligent acquisition software, the use of peptide databases for spectrum/spectrum matching using very fast scans and most importantly by increasing the dynamic range of the mass spectrometer by separately accumulating highly abundant peptides and low abundance peptides. Such advances seem possible in principle and will likely allow identification and quantification of almost all proteins in the yeast proteome in an experiment of reasonable length.

If we estimate that a particular human cell type expresses up to three times more genes than yeast, then another one or two orders of magnitude in effective sensitivity may be needed for complete coverage of a human cellular proteome. This challenge also appears to be solvable if we consider the trajectory of mass spectrometric technology improvement over the last few years.

We found that in the detected proteome our experiment already identified enough peptides to account for 25% of the primary structure of each of the proteins on average. Thus, any modifications present in this part of the proteome could in principle also have been detected and quantified. Use of a second enzyme would only double analysis time but yield much larger overall sequence coverage. At least in the case of stochiometric modifications, the chances to detect them in very complex mixtures appear quite favorable.

On the other hand, covering the proteome completely in the sense of characterizing all modifications present only on a small number of the protein population as well as all isoforms by 'brute force' approaches represents a challenge many orders of magnitude larger. This is far out of reach of currently existing technologies and will instead require targeted strategies for each of these 'subproteomes' for the foreseeable future.

## Materials and methods

### Culture growth, SILAC labeling and extract preparation

Yeast cell culture and harvesting was done as close as possible to the protocol of Ghaemmaghami *et al*. [17], in order to make results comparable. Wild-type *S. cerevisiae *cells (Y700), were grown to log-phase (OD_600 _0.7) in yeast extract peptone dextrose (YEPD) liquid medium, harvested by centrifugation for 5 minutes at 4,000 × g at 4°C, washed two times with cold H_2_O by centrifugation and immediately lysed for protein extraction. Cell membranes were disrupted by boiling in a SDS solution (50 mM Tris-HCl, pH 7.5, 5% SDS, 5% glycerol, 50 mM dithiothreitol (DTT), complete protease inhibitors cocktail (Roche, Mannheim, Germany). The total yeast lysate was centrifuged to remove cellular debris, the supernatant was transferred to a fresh tube and the protein concentration in the extract was determined by Bradford assay. For SILAC experiments, the yeast strain Y15969 (BY4742; MATα; his3D1; leu2D0; lys2D0; ura3D0; YIR034c::kanMX4), which has a lys1 gene deletion and is, therefore, an auxotroph for lysine, was purchased from EuroScarf (EuroScarf, Frankfurt, Germany). Two populations of cells were grown in yeast nitrogen base (YNB) liquid medium containing either 20 mg/l normal L-lysine or 20 mg/l L-lysine-U-^13^C_6_, ^15^N_2 _(Isotec-SIGMA, Miamisburg, OH, USA) for 10 generations, until they reached log-phase (OD_600 _0.7). Equal amounts of the normal and heavy SILAC-labeled yeast cells (as determined by OD_600 _measurement) were then mixed 1:1, harvested, washed and lysed as described above.

### 1D SDS-PAGE and in-gel digestion of yeast proteins

Proteins (100 μg) extracted from wild-type or lysine-auxotroph yeast cells were separated by 1D SDS-PAGE, using NuPAGE^® ^Novex Bis-Tris gels and NuPAGE^® ^MES SDS running buffer (Invitrogen, Carlsbad, CA, USA) according to the manufacturer's instructions. The gel was stained with Coomassie blue using Colloidal Blue Staining Kit (Invitrogen), cut in 20 slices and protein bands were excised and digested with either trypsin (Promega, Madison, WI, USA) or endoproteinase Lys-C (Wako, Osaka, Japan). Gel bands were cut into 1 mm^3 ^cubes, washed four times with 50 mM ammonium bicarbonate, 50% ethanol and incubated with 10 mM DTT in 50 mM ammonium bicarbonate for 1 hour at 56°C for protein reduction. The resulting free thiol (-SH) groups were subsequently alkylated by incubating the samples with 55 mM iodoacetamide in 50 mM ammonium bicarbonate for 1 hour at 25°C in the dark. Gels were washed two times with a 50 mM ammonium bicarbonate, 50% acetonitrile solution, dehydrated with 100% ethanol and dried in a vacuum concentrator. The gel pieces were re-hydrated with either 12.5 ng/μl trypsin (wild-type cells) or 12.5 ng/μl endoproteinase Lys-C (SILAC-labeled cells) in 50 mM ammonium bicarbonate and incubated for 16 hours at 37°C for protein digestion. Supernatants were transferred to fresh tubes, and the remaining peptides were extracted by incubating gel pieces two times with 30% acetonitrile (MeCN) in 3% trifluoroacetic acid (TFA), followed by dehydration with 100% MeCN. The extracts were combined, desalted using RP-C_18 _StageTip columns [39] and the eluted peptides used for mass spectrometric analysis.

### NanoLC-MS/MS and data analysis

All digested peptide mixtures were separated by online reversed-phase (RP) nanoscale capillary liquid chromatography (nanoLC) and analyzed by electrospray tandem mass spectrometry (ES MS/MS). The experiments were performed with an Agilent 1100 nanoflow system connected to an LTQ-FT mass spectrometer (Thermo Electron, Bremen, Germany) equipped with a nanoelectrospray ion source (Proxeon Biosystems, Odense, Denmark). Binding and chromatographic separation of the peptides took place in a 15 cm fused silica emitter (75 μm inner diameter) in-house packed with RP ReproSil-Pur C_18_-AQ 3 μm resin (Dr Maisch GmbH, Ammerbuch-Entringen, Germany).

Peptide mixtures were injected onto the column with a flow of 500 nl/minute and subsequently eluted with a flow of 250 nl/minute from 5% to 40% MeCN in 0.5% acetic acid, in a 120 minute gradient. The mass spectrometer was operated in data dependent mode to automatically switch between MS and MS/MS (MS^2^) acquisition. Survey full scan MS spectra (from m/z 300 to 1,600) were acquired in the FT-ICR with resolution R = 100,000 at m/z 400 (after accumulation to a target value of 5,000,000 in the linear ion trap). The five most intense ions were sequentially isolated and fragmented in the linear ion trap using collisionally induced dissociation at a target value of 10,000. Former target ions selected for MS/MS were dynamically excluded for 30 seconds. Total cycle time was approximately 3 seconds. The general mass spectrometric conditions were: spray voltage, 2.4 kV; no sheath and auxiliary gas flow; ion transfer tube temperature, 100°C; collision gas pressure, 1.3 mTorr; normalized collision energy using wide-band activation mode; 35% for MS^2^. Ion selection thresholds were 250 counts for MS^2^. An activation q = 0.25 and activation time of 30 ms was applied in MS^2 ^acquisitions.

### Recalibration algorithm for increased mass accuracy under space charge conditions

To boost the number of ion trap sequencing events during the online LCMS analysis, we operate the LTQ-FT in full sequencing mode (Top5), where full scan spectra are recorded in the LTQ-FT-ICR without acquiring narrow mass range (SIM) scans and with a high ion load (target of 5 × 10^6^) to maximize dynamic range. To correct for the frequency shift caused by overfilling the ICR trap, we have devised a recalibration algorithm that corrects for space charge-induced frequency errors in FT-ICR full scan spectra using already identified peptides. The algorithm is based on an iterative protein database search procedure, in which high-scoring peptides (Mascot peptide scores >35) from a first-pass database search of all acquired tandem mass spectra with loose MS tolerance (25 ppm) are used for calculating the frequency error correction. This procedure is an extension of the iterative recalibration procedure routinely used in our open source program MSQuant [40].

We compute the frequency error correction by converting the observed and calculated peptide precursor m/z values to frequencies and then determining a linear correlation between the observed and theoretical frequencies. The precursor m/z of all acquired tandem mass spectra was corrected by converting the m/z to a frequency, applying the found observed-to-theoretical linear transformation and converting the new frequency back to an m/z value. This recalibration procedure decreases the average absolute precursor mass error several fold and we achieved a final average absolute mass accuracy of 2.6 ppm. This enabled a second-pass database search with more stringent MS tolerance, in this case 10 ppm.

### Peptide identification via Mascot database search

Proteins were identified by automated database searching [41] against an in-house curated version of the yeast_orf (*S. cerevisiae*) protein sequence database. This database was complemented with frequently observed contaminants (porcine trypsin, achromobacter lyticus lysyl endopeptidase and human keratins). A 'decoy database' was prepared by sequence reversing each entry and appending this database to the forward database. Search parameters specified a MS tolerance of 10 ppm (see above) and an MS/MS tolerance at 0.5 Da and either full trypsin or Lys-C specificity as applicable, allowing for up to three missed cleavages. Carbamidomethylation of cysteine was set as a fixed modification and oxidation of methionines, amnio-terminal protein acetylation, lysine-U-^13^C_6_, ^15^N_2 _(where applicable) and N-pyroglutamate were allowed as variable modifications. Due to the high mass accuracy, the 99% significance threshold (*p *< 0.01) in the yeast database search was a Mascot score of 29. (Mascot peptide score is defined as -10 × log(p) where *p* is the probability of a false positive peptide hit.) Peptides and proteins were validated as follows. Only peptides with a length greater or equal to 5 amino acids and with a Mascot score greater or equal to 15 were considered. Peptides identifying the same sequence or sequence stretch were collapsed to one. Proteins were considered identified if 2 peptides fulfilling the above criteria mapped to their sequence and the added score of both peptides was at least 59. This protein identification criterion corresponds to 2 peptides with 99% confidence if they have the same score and an overall confidence of *p *= 0.0001 if both peptide identifications are considered independent. This stringency of identification should exclude any false positive identification in our data set. Searching a compound forward and reversed database indeed did not reveal any false positive protein identification.

## Additional data files

The following additional data are available with the online version of this paper. Additional data file 1 contains data on all peptides and proteins identified in this study.

## Supplementary Material

Additional File 1 Data on all peptides and proteins identified in this study. Click here for file

## Figures and Tables

**Table 1:** Statistics of the three large-scale mass spectrometric yeast proteomics studies

				Proteins identified		
				
Proteomic approach	Protein amount	Number of fractions	Unique peptides	1 Upep	>2 Upeps	Total
LC-MS (MudPIT)	1.4 mg	45	5,540	636	848	1,484
LC/LC-MS/MS	1.0 mg	80	7,537	513	991	1,504
GeLC-MS/MS	0.1 mg	20	20,893	NA	2,003	2,003

MudPIT refers to Washburn *et al*. [14], LC/LC-MS/MS refers to Peng *et al*. [15] and GeLC-MS/MS refers to work presented in this study. NA, not applicable; Upep, unique peptide.
